# Brain Structural and Functional Abnormalities Associated with Acute Post-Traumatic Headache: Iron Deposition and Functional Connectivity

**DOI:** 10.21203/rs.3.rs-4165756/v1

**Published:** 2024-03-28

**Authors:** Simona Nikolova, Catherine Chong, Jing Li, Teresa Wu, Gina Dumkrieger, Katherine Ross, Amaal Starling, Todd J. Schwedt

**Affiliations:** Mayo Clinic; Mayo Clinic; Georgia Tech; Arizona State University; Mayo Clinic; Phoenix VA Health Care System; Mayo Clinic; Mayo Clinic

**Keywords:** T2*, imaging, post traumatic headache, mild traumatic brain injury, iron, magnetic resonance imaging, structural, anatomical, functional connectivity

## Abstract

**Background:**

The purpose of this study was to interrogate brain iron accumulation in participants with acute post-traumatic headache (PTH) due to mild traumatic brain injury (mTBI), and to determine if functional connectivity is affected in areas with iron accumulation. We aimed to examine the correlations between iron accumulation and headache frequency, post-concussion symptom severity, number of mTBIs and time since most recent TBI.

**Methods:**

Sixty participants with acute PTH and 60 age-matched healthy controls (HC) underwent 3T magnetic resonance imaging including quantitative T_2_* maps and resting-state functional connectivity imaging. Between group T_2_* differences were determined using T-tests (p < 0.005, cluster size threshold of 10 voxels). For regions with T_2_* differences, two analyses were conducted. First, the correlations with clinical variables including headache frequency, number of lifetime mTBIs, time since most recent mTBI, and Sport Concussion Assessment Tool (SCAT) symptom severity scale scores were investigated using linear regression. Second, the functional connectivity of these regions with the rest of the brain was examined (significance of p < 0.05 with family wise error correction for multiple comparisons).

**Results:**

The acute PTH group consisted of 60 participants (22 male, 38 female) with average age of 42 ± 14 years. The HC group consisted of 60 age-matched controls (17 male, 43 female, average age of 42 ± 13). PTH participants had lower T_2_* values compared to HC in the left posterior cingulate and the bilateral cuneus. Stronger functional connectivity was observed between bilateral cuneus and right cerebellar areas in PTH compared to HC. Within the PTH group, linear regression showed negative associations of T_2_* and SCAT symptom severity score in the left posterior cingulate (p = 0.05) and with headache frequency in the left cuneus (p = 0.04).

**Conclusions:**

Iron accumulation in posterior cingulate and cuneus was observed in those with acute PTH relative to HC; stronger functional connectivity was detected between the bilateral cuneus and the right cerebellum. The correlations of decreased T_2_* (suggesting higher iron content) with headache frequency and post mTBI symptom severity suggest that the iron accumulation that results from mTBI might reflect the severity of underlying mTBI pathophysiology and associate with post-mTBI symptom severity including PTH.

## Background

Iron is detected with magnetic resonance imaging through the use of susceptibility weighted imaging sequences, including T_2_* sequences [1–3]. Decreased T_2_* signal has been shown to associate with increased iron accumulation [2, 4]. T_2_* changes have been found in neurological disorders such as Parkinson’s Disease and have been linked to cognitive decline and aging [5–7].

Iron deposits have been reported in areas of pain processing and deep brain structures of individuals with migraine [8–13]. Iron accumulation in the periaqueductal gray matter and anterior cingulate has been shown to correlate with duration of migraine and number of migraine episodes [10–12]. Recent studies suggest that iron deposits play a role in migraine chronification [8, 9]. T_2_* changes have been observed in longitudinal studies of migraine treatment, suggesting that effective migraine treatment impacts brain iron accumulation [14]. There are fewer investigations into iron concentrations among participants with post traumatic headache (PTH). A study has shown that PTH is associated with increased iron deposits in cortical, occipital, hippocampal and brainstem regions [15].

To interrogate acute PTH pathophysiology, this study aimed to identify brain areas with T_2_* differences in PTH patients relative to healthy controls (HC) and to determine if these areas with structural differences demonstrate altered resting-state functional connectivity. Associations between iron deposition and clinical characteristics were investigated to gain insights into relationships between abnormal brain structure with mTBI and acute PTH characteristics.

## Methods

### Subject Enrollment

This study was approved by the Mayo Clinic and Phoenix VA Institutional Review Boards. Study participants were between 18 and 65 years of age. PTH participants were enrolled from Mayo Clinic Arizona and the Phoenix VA Health Care System and HCs were recruited through advertisements and word-of-mouth. All participants completed an informed consent process and signed informed consent forms. Data were collected over a three-year period between 2020 and 2023. Participants were diagnosed with acute PTH due to mTBI using the ICHD-3 criteria [16]. PTH participants were enrolled between the day of their injury until 59 days post mTBI. Participants completed questionnaires and structured interviews that collected data on patient demographics, current and prior mTBI characteristics, and headache characteristics [17]. Exclusion criteria for HC and PTH included gross anatomical abnormalities on brain MRI, history of severe psychiatric disorder as assessed by the principal investigator, such as but not limited to schizophrenia and bipolar disorder, history of speech disorders, pregnancy, and history of moderate or severe TBI. HC participants were excluded if they had history of mTBI or migraine. HC participants with tension-type headaches on three or fewer days per month were not excluded.

### Imaging Acquisition

Neuroimaging was performed on a 3T Siemens scanner (Siemens Magnetom Skyra, Erlangen, Germany) at Mayo Clinic Arizona. A 20-channel head and neck coil was used for all imaging. T_1_-weighted images were acquired for anatomical reference using a magnetization prepared rapid image acquisition gradient echo (MPRAGE) sequence with 1mm isotropic resolution with echo time (TE = 3.03 ms), repetition time (TR = 2400 ms), and flip angle (FA = 8 degrees) covering 160 × 256 × 256 mm^3^. To obtain T_2_* maps 12 T_2_-weighted gradient echo (GRE) magnitude images were used with varying echo times (TE = 2.81, 5.71, 8.06, 10.46, 12.93, 15.4, 17.86, 29.78, 42.34, 54.9, 67.46 and 80 ms). Each GRE was a stacked 2D axial image with FA of 60 degrees, TR of 3200ms, in-plane resolution of 0.94 × 0.94 mm, slice thickness of 4 mm and FOV of 240×240×132 mm^3^.

Two five-minute runs of resting state blood oxygen level dependent (BOLD) imaging with TE of 27 ms, TR of 2500 ms, FOV 64×64×64 mm^3^, FA of 90 degrees, and voxel size 4×4×4 mm were collected while participants relaxed with their eyes closed. For each participant, the T_1_ and T_2_ imaging sequences were reviewed by a neuroradiologist, and imaging was excluded from further analyses if abnormal brain imaging findings were present.

### Image Postprocessing

Data pre-processing was done using SPM12 software (Wellcome Department of Cognitive Neurology, Institute of Neurology, London, UK) in conjunction with MATLAB version R2019b (Mathworks, Natick, MA, USA). T_2_* maps were smoothed with a 6 mm kernel and resampled to match the T_1_-weighted images. The T_1_-weighted images were used to normalize to Montreal Neurological Institute (MNI) space along with the co-registered T_2_* to yield 1mm isotropic resolution maps. A 4 mm dilated mask was used to remove cerebrospinal fluid (CSF) contamination.

Functional images were motion corrected, realigned to the first image of the set, coregistered to the anatomical T_1_-weighted image, realigned to the average MNI template, and smoothed using an 8 mm FWHM Gaussian kernel using SPM12. Further post processing in AFNI 3dTproject included band pass filtering (0.01–0.1 Hz) after removal of nuisance signals from framewise displacement, cerebrospinal fluid signal, and linear drift. The first five frames were excluded to allow the signal to reach steady state. Frames with excess of 2 mm motion were also removed from the analysis. Region of interest to brain correlation maps were calculated using MATLAB. Regions of significant T_2_* difference between PTH and HC were used to select the regions of interest for the functional connectivity analysis. Left and right hemisphere clusters were interrogated separately. The Pearson correlation coefficients were calculated between the region of interest and the rest of the brain using in-house software written in MATLAB. The correlation maps were Fisher transformed to Z-scores for group comparisons.

### Statistical Analysis

T_2_* differences between 60 PTH participants and 60 age-matched HC were evaluated in MNI space using a t-test within SPM12 (https://www.fil.ion.ucl.ac.uk/spm/software/spm12/) compensating for age and sex. Cluster analysis was performed in SPM 12 using uncorrected p < 0.005 and volume threshold of 10 mm^3^ (10 voxels) to interrogate T_2_* differences in PTH compared to HC. Cluster labelling was assisted with automated anatomical atlas ROI_MNI_V7.

The average T_2_* values from regions with significant T_2_* differences were included in a linear model to assess association with clinical variables within the PTH group. The clinical variables examined were number of lifetime mTBIs, time since most recent mTBI, the Sport Concussion Assessment Tool (SCAT) symptom severity checklist score, and headache frequency. Headache frequency was calculated as the percentage of days with headache since mTBI.

The regions with significant T_2_* differences between PTH and HC were used as seeds for a for full brain resting state functional connectivity correlation analysis. A full factorial analysis was conducted in SPM12 with two factors: group factor with two levels for PTH and HC and region of interest factor with the number of levels equal to the number of regions of interest used, as well as covariates to compensate for age and sex. Group differences for each region of interest were examined with a F-statistic with post hoc t-tests used to assess direction of the effect. Significance for group differences was set to p < 0.05 with family wise error correction for multiple comparisons.

## Results

A total of 120 participants were included in the analysis. Participant demographics, headache characteristics, and mTBI characteristics are shown in Table 1. The acute PTH group consisted of 60 participants (22 male, 38 female) with average age of 42 years (SD = 14, range from 19 to 70). The HC group consisted of 60 age-matched controls (17 male, 43 female) with an average age of 42 (SD = 13, range from 21 to 71). Age matching (PTH-HC) had an average difference of 0.1 years (SD = 2, range – 3.6 to 5.3 years).

The symptoms included in the ICHD-3 diagnostic criteria for primary headaches were used to classify the PTH phenotypes: 35 participants with PTH had migraine-like headache phenotypes, 8 had probable migraine-like phenotypes, 14 had tension-type-like headache phenotypes, 1 had probable tension type-like headache phenotype, and 2 participants’ PTH phenotypes were not classifiable.

Forty-five percent of all PTH participants suffered a mTBI due to a fall, 30% due to motor vehicle accident, 12% due to a fight, 7% hit against object, 5% due to a sport injury, and 2% hit by object. Forty-six percent of PTH participants had one lifetime mTBI, 17% had two mTBIs, 16% had three, 5% had four, and 16% had 5 or more lifetime TBIs. For all participants, the number of reported days between mTBI and imaging ranged from 4 to 50 days with an average of 25 days (SD = 15 days). Twenty-seven participants had PTH onset immediately following injury, 23 had PTH starting within one day of the mTBI, and ten had PTH onset of > 1 day post mTBI but prior to 7 days. PTH participants had headaches on an average of 81% of the days since their TBI (SD = 28% with a range from 7–100%). Fifty-five percent of PTH participants had continuous headaches.

### T_2_* Imaging Results

**Figure 1:**
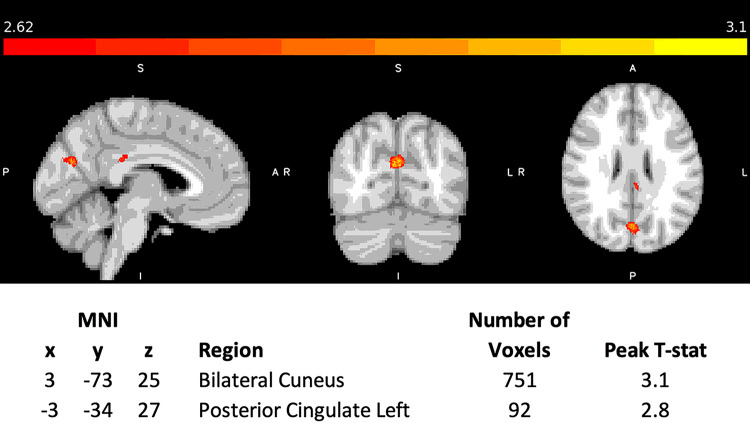
Whole brain analysis of cluster results of T_2_* reduction in participants with post-traumatic headache compared to age-matched healthy controls. 3D T-statistic of T_2_* decreases in PTH participants compared to HC are shown in standardized MNI space. The two y-clusters surviving uncorrected p < 0.005 with cluster threshold of 10 mm^3^ (10 voxels) are the left posterior cingulate and the bilateral cuneus. The coordinates (x,y,z) in MNI space of the cluster centers, cluster volume and the corresponding anatomical regions are shown. maximum T-statistic at the cluster center is provided.

No increases of T_2_* were detected in PTH participants compared to healthy controls, whereas lower T_2_* values (suggesting increased iron) were observed in the left posterior cingulate and bilateral cuneus as shown in Fig. 1. Since the cuneus results were bilateral, the left and right sides were investigated separately in a secondary analysis to account for laterality.

### T_2_* Linear Regression of Headache and mTBI Characteristics

For those regions that had significant T_2_* differences between PTH and HC, significant negative associations were found between SCAT symptom severity score and left posterior cingulate (p = 0.05) and between left cuneus and headache frequency (p = 0.04).

#### Functional Connectivity Differences in Brain Regions with T_2_* decreases.

In PTH participants, the regions with decreased T_2_* in PTH compared to HC, the posterior cingulate cortex and cuneus, were investigated for differences in resting state functional connectivity. Significantly stronger functional connectivity was observed between the right cuneus with right cerebellum and the left cuneus with right cerebellum amongst those with PTH compared to HC.

## Discussion

It has previously been shown that iron deposition may serve as a biomarker for migraine and for duration and frequency of migraine attacks [10–12]. The effect of iron deposition in PTH hasn’t been widely investigated, but the findings from our study suggest that iron deposition might also serve as a biomarker for PTH frequency. In a previous study, we observed T_2_* decreases associated with iron accumulation in twenty PTH participants compared to age matched HCs [15]. Nineteen of the twenty abovementioned PTH participants were included in the current study, along with seventeen of the HCs. The current, larger study reports regions with T_2_* decreases consistent with previous findings [15] with more conservative thresholds used for determining significance. The current study found decreased T_2_* in the bilateral cuneus and left posterior cingulate. The cuneus is involved in visual processing, as well as having roles in working memory and cognitive control. The posterior cingulate, along with precuneus, angular gyrus and medial prefrontal cortex is a core region of the default mode network (DMN) [18–22]. Our results suggest that brain iron accumulation serves as a biomarker of PTH and mTBI burden and might provide insights into mechanisms underlying PTH and other symptoms following mTBI.

Our results suggest that T_2_* decreases may play a role in the disruption of cerebellar functional networks, previously shown in PTH after mTBI, and consistent with the hypothesis of hypersensitivity to pain and disruption of cognitive control networks [23]. Specifically, we saw increased functional connectivity from the bilateral cuneus to the cerebellum. We speculate that altered structure of the cuneus and its connectivity with the cerebellum could be associated with abnormal eye movements that often occur following mTBI, such as abnormalities in saccades, smooth pursuit, and vergence [24–26]. Unfortunately, we did not measure oculomotor dysfunction in our study.

Prior studies have demonstrated atypical structure and connectivity of DMN regions amongst those with mTBI and amongst those with PTH [23, 27, 28]. In our study, abnormal T_2_* was identified in the posterior cingulate, an important hub of the DMN. Niu et al. reported disruption in the connectivity between the DMN with the periaqueductal gray amongst those with acute PTH due to mTBI which could signify impaired pain modulation following mTBI and which was a strong predictor of PTH persistence at three months post injury [23, 27]. Impaired pain modulation leading to oxidative stress could be a possible mechanism resulting in increased T_2_*.

Zhou et al. showed reduced connectivity in the posterior cingulate and parietal regions and increased frontal connectivity in the medial prefrontal cortex in patients with mTBI relative to HC. In this study, we also observed opposing changes in functional connectivity with some connections being stronger and others being weaker. Zhou et al. also showed that the posterior connectivity correlated positively with neurocognitive dysfunction while the frontal connectivity was negatively correlated to post-traumatic symptoms such as symptoms of postconcussion syndrome. In this study, we did not interrogate functional connectivity correlations to headache severity, but we did show that headache severity correlated negatively with iron burden in the left posterior cingulate and with headache frequency in the left cuneus.

Iron accumulation in the two regions found in this study correlated with measures of headache frequency and post-mTBI symptom severity. We found negative associations of headache frequency with T_2_* in the left cuneus, one region previously identified to associate with headache frequency [19, 29, 30]. Negative association between headache frequency and decrease of gray matter volume in the cuneus was reported by Neeb et al. [30] in migraine groups compared to HCs. This finding suggests a link between pain and brain plasticity, independent of injury. The negative associations with headache frequency suggest that there may be an accumulation of iron due to recurrent headaches.

### Limitations

T_2_* decrease is assumed to relate to iron accumulation, but that decrease may be due to hemorrhage, venous blood, calcification, or changes in tissue water concentration. Future work will evaluate these contrast contributions further using phase information from quantitative susceptibility imaging.

In this study, regions with T_2_* decreases consistently showed cerebellar connectivity disruption yet no T_2_* differences in the cerebellum are reported. The field of view of our T_2_* sequence consistently cropped the cerebellum and data from this region could not be reported. Future studies should expand the field of view to contain the cerebellum.

The exploratory nature of this study created numerous statistical comparisons between groups, and within group associations. No corrections for multiple comparisons were made for the primary analysis to identify regions with T_2_* decreases in PTH in line with other exploratory whole brain studies [31]. Type I errors were mitigated through a cluster forming threshold of 10 voxels for all reported significant clusters. A correction for multiple comparisons was applied to the secondary full brain analysis for connectivity changes.

The temporal dynamics of iron accumulation after mTBI has not been thoroughly investigated and is not well understood. Time since mTBI was included in the multiple linear regression analysis in the current study when investigating the effect of headache characteristics, but it is possible that non-linear models would be more appropriate.

## Conclusions

Decreased T_2_* values, suggestive of increased iron accumulation, were found in the left posterior cingulate cortex and bilateral cuneus amongst those with acute PTH attributed to mTBI compared to HC. Stronger functional connectivity was observed between the bilateral cuneus and the right cerebellum. In the regions with decreased T_2_* in PTH compared to HC, headache frequency, and SCAT symptom severity scores correlated with iron accumulation, suggesting that the presence of iron is associated with greater mTBI and PTH burden.

## Figures and Tables

**Table 1:** Participant demographics and post traumatic headache clinical variables and phenotypes. Time since mTBI is the number of days between the date of mild traumatic brain injury and the baseline research visit. Headache frequency is reported as the percent number of days since mTBI with headache. Time from mTBI to PTH is the number of hours between the brain injury and headache onset. Values reported as mean ± standard deviation. Abbreviations: mTBI = mild traumatic brain injury, PTH = post traumatic headache, HC = healthy controls.

	PTH (n = 60)	HC (n = 60)
Age (years)	41.7 ± 13.7	41.5 ± 13.4
Sex (male/female)	22/38	17/43
Headache frequency (%)	81 ± 28	N/A
Number of lifetime mTBIs	2.4 ± 1.9	N/A
Time since most recent mTBI (days)	25 ± 15	N/A
SCAT symptom checklist score	29 ± 23	N/A
Time from mTBI to PTH (hours)	17 ± 47	N/A
PTH Phenotypes		
Migraine	35	N/A
Probable migraine	8	N/A
Tension type	14	N/A
Probable Tension type	1	N/A
Not Classifiable	2	N/A

## Data Availability

Deidentified data from this study can be made available upon request following Mayo Clinic sharing standards. Analytic code used to conduct the statistical analyses presented in this study are freely available in a public repository identified in the manuscript (SPM12). The datasets used and/or analyzed during the current study are available from the corresponding author on reasonable request.

## Abbreviations

HC: Healthy Control
PTH: Post traumatic headache
mTBI: mild traumatic brain injury
DMN: default mode network
SCAT: sport concussion assessment tool
MPRAGE: magnetization prepared rapid image acquisition gradient echo
GRE: Gradient echo
BOLD: Blood-oxygen-level-dependent
GLM: general linear model
MNI: Montreal Neurological Institute
ROI: region of interest
